# A genetic variant in *SLC30A2* causes breast dysfunction during lactation by inducing ER stress, oxidative stress and epithelial barrier defects

**DOI:** 10.1038/s41598-018-21505-8

**Published:** 2018-02-23

**Authors:** Sooyeon Lee, Yandong Zhou, Donald L. Gill, Shannon L. Kelleher

**Affiliations:** 1 Department of Cellular and Molecular Physiology, Penn State Hershey College of Medicine, Hershey, PA 17033 USA; 2 Department of Pharmacology, Penn State Hershey College of Medicine, Hershey, PA 17033 USA; 3 Department of Surgery, Penn State Hershey College of Medicine, Hershey, PA 17033 USA; 4 Present Address: Department of Medicine, Stanford University, Stanford, CA 94305 USA; 5 Present Address: Department of Biomedical and Nutritional Sciences, University of Massachusetts Lowell, Lowell, MA 01854 USA

## Abstract

*SLC30A2* encodes a zinc (Zn) transporter (ZnT2) that imports Zn into vesicles in highly-specialized secretory cells. Numerous mutations and non-synonymous variants in ZnT2 have been reported in humans and in breastfeeding women; ZnT2 variants are associated with abnormally low milk Zn levels and can lead to severe infantile Zn deficiency. However, ZnT2-null mice have profound defects in mammary epithelial cell (MEC) polarity and vesicle secretion, indicating that normal ZnT2 function is critical for MEC function. Here we report that women who harbor a common ZnT2 variant (T^288^S) present with elevated levels of several oxidative and endoplasmic reticulum (ER) stress markers in their breast milk. Functional studies *in vitro* suggest that substitution of threonine for serine at amino acid 288 leads to hyperphosphorylation retaining ZnT2 in the ER and lysosomes, increasing ER and lysosomal Zn accumulation, ER stress, the generation of reactive oxygen species, and STAT3 activation. These changes were associated with decreased abundance of zona occludens-1 and increased tight junction permeability. This study confirms that ZnT2 is important for normal breast function in women during lactation, and suggests that women who harbor defective variants in ZnT2 may be at-risk for poor lactation performance.

## Introduction

Zinc (Zn) is an essential ion required by 10% of the eukaryotic proteome that plays a vital role in over 300 cellular processes (e.g. transcription, translation, enzyme activity and intracellular signaling) and functions (e.g. proliferation, differentiation, polarity, apoptosis, and autophagy). As a result, tight regulation of intracellular Zn transport is critical for normal cell function, which is regulated through the expression, sub-cellular localization and function of members of two gene families of solute transporters-*SLC39A* and *SLC30A*. Recent studies have identified genetic variation in these gene families that underlie various disease conditions in humans, including severe acquired Zn deficiency^1–5^, diabetes^6,7^ and schizophrenia^8^. However, the molecular defects that contribute to many of these pathologies are still under investigation.

Expression of *SLC30A2* (ZnT2) is restricted to secretory cells, such as acinar pancreatic cells, prostate epithelial cells, placental trophoblasts, Paneth cells, and mammary epithelial cells (MECs)^9,10^. ZnT2 consists of six transmembrane domains with cytoplasmic N- and C-termini^11^ that contain numerous regulatory domains^12,13^, and functions as a homo- or heterodimer to transport Zn into vesicles^4,14,15^. Because of its importance during lactation, most information regarding the role and regulation of ZnT2 comes from studies in the mammary gland. In non-secreting MECs, ZnT2 transports Zn from the cytoplasm into mitochondria^13^ and vesicles^14^. During lactation, the lactogenic hormone prolactin transcriptionally up-regulates ZnT2 expression through the binding of STAT5 to two GAS elements in the *SLC30A2* promoter^16^, and post-translationally re-localizes ZnT2 to secretory vesicles to motivate Zn secretion into milk, partially through the ubiquitination of two lysine residues (K^4/6^) in the N-terminus^17^. Moreover, we recently reported that loss of ZnT2 function in lactating ZnT2-null mice results in cytoplasmic Zn accumulation in MECs, and leads to impaired mammary gland architecture and defects in MEC polarity, which is associated with an overall loss of secretory capacity, low milk volume and early neonatal death^18,19^. In addition, recent studies show that ZnT2 is important for breast remodeling during involution. Treatment of MECs with the pro-involution signal tumor necrosis factor alpha (TNFα) *in vitro* dephosphorylates ZnT2 at S^296^, which enhances binding of adaptor protein-3 (AP3) to a conserved dileucine motif (L^293–295^) proximal to this phosphorylation site^12^. AP3 binding re-localizes ZnT2 to lysosomes driving lysosomal Zn import and activating lysosomal-mediated cell death. These observations have been recapitulated *in vivo* as intramammary injection of TNFα rapidly leads to lysosomal-mediated cell death and precocious involution^20^. Collectively, these studies reveal the complex and multifactorial role of ZnT2, and indicate that it plays key roles in mammary gland function that go well-beyond the secretion of Zn into milk.

The importance of understanding ZnT2 function reflects the fact that thus far, eight missense mutations have been identified in human *SLC30A2* (H^54^R, G^87^R, W^152^R, G^280^R, S^296^L, T^312^M, R^340^C and E^355^Q) that lead to pathologically low breast milk Zn concentrations (~50–95% reduction) and severe Zn deficiency in breastfed infants^2–5^. This disorder, known as “transient neonatal Zn deficiency,” can result in immunoinsufficiency, cognitive delays, growth faltering and mortality if not diagnosed early. In addition, public archives of genome wide association studies (e.g., dbSNP) have compiled data on numerous non-synonymous genetic variants in *SLC30A2* in humans, and translational studies have shown that many of these variants compromise ZnT2 function and lead to sub-optimal health outcomes^1,21^. For example, we and others showed that expression of ZnT2 variants *in vitro* can result in aberrant sub-cellular Zn transport^1,4^, cytotoxic Zn accumulation^1,22^ and alterations in cell cycle^1^. Of all the ZnT2 variants that have thus far been identified, a threonine to serine substitution at amino acid 288 (T^288^S) in the C-terminus of ZnT2 is most common, and was detected in 18% of breastfeeding women as both compound and simple heterozygous substitutions^1^. In addition to abnormally low milk Zn concentration, women who are heterozygous for the S^288^ variant also have elevated milk sodium levels^1^, a classic hallmark of tight junction impairment, breast dysfunction and premature wearning^23–27^. Taken together this suggests that women who harbor select ZnT2 variants may be at risk for sub-optimal lactation.

In this report, we present evidence that women who harbor the most common genetic variant in *SLC30A2* that has been identified thus far (T^288^S) secrete molecular factors into their breastmilk that suggest they are suffering from breast dysfunction. To further explore this possibility, we determined that the S^288^ variant was hyperphosphorylated and retained in the ER and lysosomes, leading to increased ER and lysosomal Zn accumulation, ER and oxidative stress, defects in tight junction and paracellular barrier formation, and precocious STAT3 activation in cultured MECs. These results indicate that expression of ZnT2 variants in breastfeeding women have important consequences on sub-cellular Zn pools and the molecular regulation of MEC function, which may lead to precocious breast remodeling and poor lactation performance.

## Results and Discussion

### Mothers harboring the S^288^ variant have markers of oxidative stress and breast dysfunction in their breast milk

**Figure 1:**
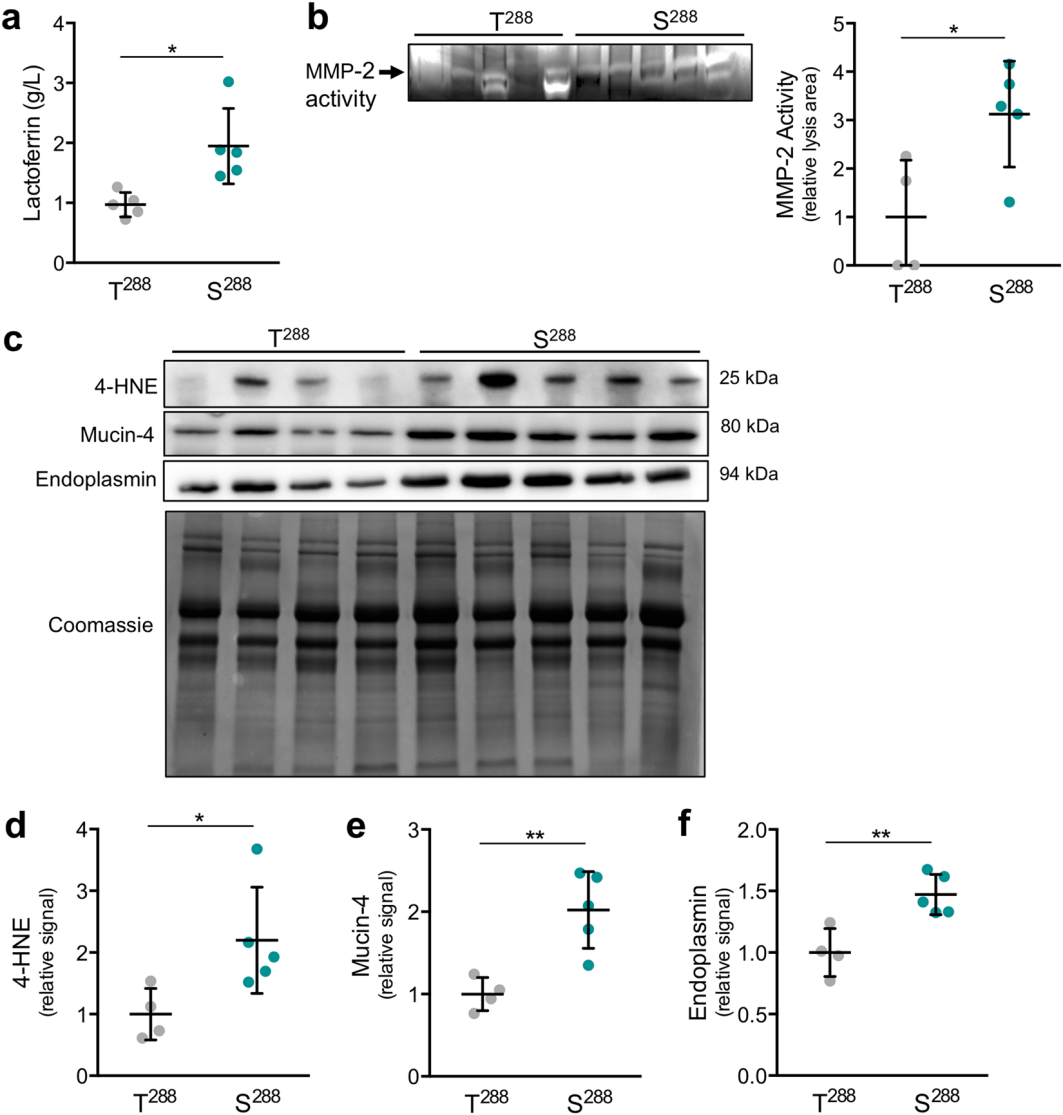
Markers of breast dysfunction and oxidative stress in breast milk from women harboring wild-type ZnT2 (T^288^) or the ZnT2 variant (S^288^). (**a**) Measurement of lactoferrin concentration in breast milk from women harboring two wild-type ZnT2 alleles (T^288^) and women harboring the ZnT2 variant (S^288^). Milk lactoferrin concentration was measured by ELISA. Data represent mean milk lactoferrin concentration (g/L) ± SD from n = 5 samples/genotype. (**b**) Evaluation of MMP-2 activity in breast milk from women harboring T^288^ or S^288^. MMP-2 activity was determined by gelatin zymography (arrow); data represent mean gelatin lysis area (clear bands) relative to T^288^ ± SD from n = 4–5 samples/genotype. Cropped gel is displayed and full-length gel can be found in Supplementary Fig. S2a. (**c**) Representative immunoblots of oxidative stress markers (4-HNE, mucin-4 and endoplasmin) in a fixed volume (5 µL) of breast milk from women harboring T^288^ or S^288^. A replicate gel was stained with Coomassie Blue as a loading control (bottom panel). Cropped blots are displayed and full-length blots can be found in Supplementary Fig. S2b–d. Quantification of relative protein abundance of (**d**) 4-HNE, (**e**) mucin-4 and (**f**) endoplasmin. Data represent mean signal intensity normalized to T^288^ ± SD from n = 4–5 samples/genotype; p < 0.05*, p < 0.01**.

A threonine to serine substitution at amino acid 288 (S^288^) in ZnT2 was previously detected in 18% of breastfeeding women in a previous study, and the milk from women who were simple heterozygotes for this variant contained significantly elevated sodium levels^1^. Milk sodium levels normally increase at the time of involution^28,29^, and elevated milk sodium levels have been observed in women with mastitis and breast inflammation^23–26^, resulting from the deterioration of intercellular junctions driven by oxidative stress^30–32^. To investigate the consequence of harboring S^288^ on lactation outcomes in breastfeeding women, we first assessed milk macronutrient concentrations (protein, lactose and fat) and found that they were similar to that observed in the milk of women harboring two wild-type alleles (T^288^) (Supplementary Table S1). This indicates that milk energy density was similar, and suggests that overt health consequences from consuming inadequate/excessive energy would not be expected in their infants. However, because milk sodium was elevated and is associated with oxidative stress in breast remodeling^24,25^ and premature weaning^27^, we speculated that more subtle problems with lactation performance might exist. Therefore, we analyzed the milk for several molecular factors that may reflect poor lactation performance and have previously been associated with oxidative stress, such as elevated lactoferrin levels and matrix metalloproteinase 2 (MMP-2) activity^33,34^. Lactoferrin is an iron-binding protein that protects against oxidative stress by preventing the Fenton reaction, which converts hydrogen peroxide into highly reactive hydroxyl radicals^35^, or by directly scavenging hydroxyl radicals^36^. Elevated milk lactoferrin has been suggested as a marker of poor lactation performance or breast dysfunction, as increased lactoferrin expression in MECs parallels the presence of pathogens, cytokines, and the activation of breast remodeling^37^. Activation of MMP-2 is a crucial event that occurs in response to damage induced by oxidative stress^38^ or directly by the reaction of oxygen radicals with thiol groups within MMP-2^39^. Moreover, oxidative stress activates MMP-2 to facilitate invasion and metastasis, thus MMP-2 activity increases during breast remodeling^31^ and in breast tumors^40,41^. Consistent with these reports, we found that milk from women harboring S^288^ had significantly greater lactoferrin and MMP-2 activity compared with women harboring T^288^ (Fig. 1a and b). This provides evidence that expression of the S^288^ variant is associated with oxidative stress and dysfunction in the breast during lactation. To confirm this, we measured several key oxidative stress markers; 4-hydroxynonenal (4-HNE), a product of lipid peroxidation^42^, mucin-4, an adhesive glycoprotein that is upregulated in response to oxidative stress^43^, and endoplasmin, an ER-specific chaperone that increases in response to ER and oxidative stress^44^. We found that 4-HNE, mucin-4 and endoplasmin were all significantly higher in the breastmilk of women harboring S^288^ compared with women harboring two wild-type alleles (Fig. 1c–f). To our knowledge, this is the first report to suggest that mucin-4 and endoplasmin may be useful as stress markers in breast milk. Taken together, our data indicate that women harboring the S^288^ variant have detectable levels of novel biological factors in their breast milk that suggests enrichment in oxidative and ER stress in their breast tissue and breast dysfunction. It is important to note that this was a cross-sectional study of women who had been breastfeeding for ~4 months, and information on feeding patterns (e.g., exclusive versus partial breastfeeding) and infant health was not collected. These results argue in favor of conducting detailed clinical studies to determine effects of ZnT2 variants on milk volume, lactation outcomes and infant health.

### S^288^ expression leads to ER Zn accumulation and induces ER stress in MECs

**Figure 2:**
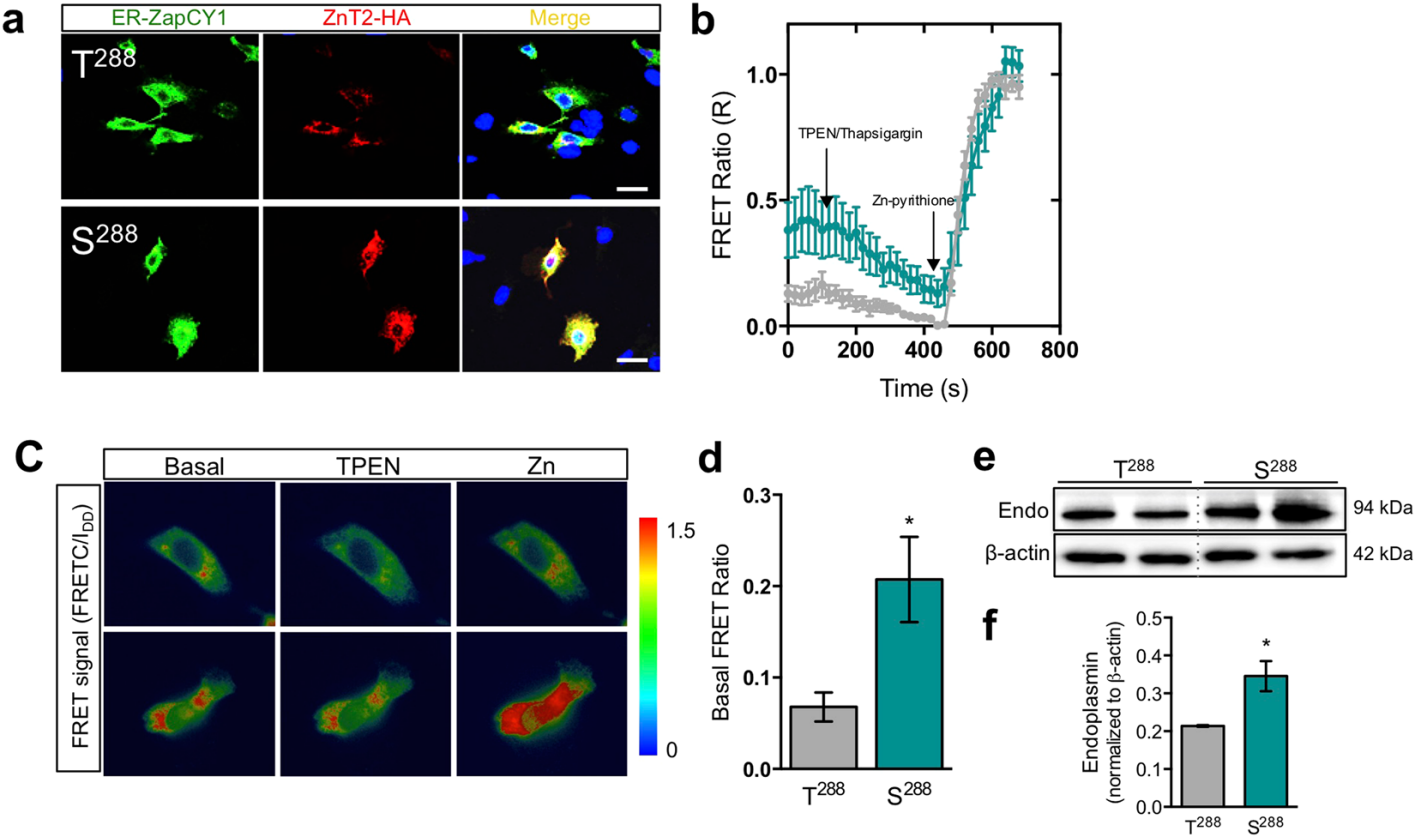
Ectopic expression of the S^288^ variant of ZnT2 is retained in the ER, accumulates Zn in ER, and induces ER stress. (**a**) Representative confocal images of ER-ZAPCY1 (green) and ZnT2-HA (red) in MECs transfected to express wild-type ZnT2 (T^288^) and the ZnT2 variant (S^288^). Merged images (yellow) illustrate co-localization of ER-ZAPCY1 and ZnT2. Nuclei were counterstained with DAPI (blue). Note robust co-localization of ER-ZAPCY1 and ZnT2-HA in MECs expressing S^288^ (Pearson’s coefficient = 0.87), indicating ER localization of S^288^ compared with MECs expressing T^288^ (Pearson’s coefficient = 0.34; scale bar, 25 µm). (**b**) Representative FRET analysis demonstrating the changes in FRET ratio (R) of ER-ZAPCY1 in MECs expressing T^288^ or S^288^ treated with TPEN (100 µM) and thapsigargin (2 µM; R_min_) followed by zinc pyrithione (100 µM; R_max_), n = 10–14 cells/genotype, from four independent experiments. (**c**) Representative pseudocolored FRET signal images of ER-ZAPCY1 in MECs expressing T^288^ or S^288^ at rest (Basal), after TPEN (100 µM) + thapsigargin (2 µM; TPEN) treatment, in each case followed by zinc pyrithione (100 µM; Zn) treatment (scale bar, 10 µm). (**d**) Quantification of basal FRET ratio in MECs expressing T^288^ or S^288^. Data represent mean FRET ratio at basal levels ± SEM, n = 10–14 cells/genotype, from four independent experiments; p < 0.05*. (**e**) Representative immunoblot of endoplasmin (Endo) in total lysates from MECs expressing T^288^ or S^288^ treated with Zn. β-actin served as a loading control. Dotted lines indicate spliced sections obtained from a single blot; representative samples (n = 2/group) were selected for publication. Spliced blots are displayed and full-length blots can be found in Supplementary Fig. S3a,b. (**f**) Quantification of endoplasmin expression. Data represent mean endoplasmin expression normalized to β-actin ± SD, n = 6 samples/genotype, from three independent experiments; p < 0.05*.

Lactation normally upregulates ER stress-related genes in the mammary gland^45^ to meet the high metabolic demands of milk production and secretion^46–49^. Enhanced ER stress can lead to an increase in unfolded and misfolded proteins, which activates the unfolded protein response (UPR) as a regulatory mechanism to restore ER homeostasis and maintain lactation^50,51^. However, when unrestrained, ER stress induces expression of ER chaperones like endoplasmin, to increase ER capacity and also inhibit protein translation to reduce ER load, which can lead to lactation failure. Because we found evidence of enhanced ER stress in women harboring S^288^, and previous studies from our lab showed that the S^288^ variant is retained in the ER in MECs^1^, we predicted that expressing S^288^ in cultured MECs would increase ER Zn levels and ER stress, ultimately leading to increased ROS and oxidative stress. To test this directly, we used ratiometric imaging of ER-ZAPCY1, a Zn-responsive sensor that is targeted to the ER that upon Zn binding, inducing a conformational change that leads to an increase in fluorescence resonance energy transfer (FRET)^52^. To first verify that ER-ZAPCY1 localizes appropriately to the ER in our cultured MEC system, we co-localized ER-ZAPCY1 with the ER marker calnexin to confirm that this Zn sensor was indeed targeted correctly to the ER in MECs (Supplementary Fig. S2). Next, we co-transfected MECs with either T^288^ or S^288^ together with ER-ZAPCY1, and localization was visualized by confocal microscopy (Fig. 2a). We found that while T^288^ was minimally co-localized with ER-ZAPCY1 (Pearson’s coefficient: 0.34), the S^288^ variant showed strong co-localization with ER-ZAPCY1 (Pearson’s coefficient: 0.87), confirming that S^288^ is retained in the ER. To determine if the retained variant was capable of transporting Zn into the ER, we used FRET analysis and found that MECs expressing the S^288^ variant had a significantly greater FRET ratio compared to MECs expressing T^288^ (Fig. 2b–d). It is interesting to note that only basal ER Zn levels were elevated in MECs expressing S^288^ and that the rate of Zn transport into the ER was similar, at least within the experimental conditions explored. One would predict that because there is substantially greater S^288^ retained within the ER, then the rate of Zn accumulation should also be greater. As this was not the case, it suggests that the S^288^ variant may have reduced Zn transporting activity, similar to our previous observations of several other ZnT2 mutants (K^66^N, Q^71^H, D^103^E, and T^312^K)^1^, and also to that observed by Golan and colleagues (G^280^R, E^355^Q and T^312^M)^53^. Further studies are required to define the precise mechanism(s) through which the S^288^ substitution confers defects in Zn transporting activity. A consequence of Zn accumulation in the ER is ER stress^54^. Consistent with our observation that women harboring S^288^ had greater endoplasmin levels in their milk, we found that MECs expressing S^288^ had significantly greater expression of endoplasmin compared to MECs expressing T^288^ (Fig. 2e and f). Collectively, these results indicate that retention of the S^288^ variant in the ER directly increases ER Zn levels and ER stress, which may lead to breast dysfunction and poor lactation performance^54^.

### MECs expressing S^288^ have increased oxidative stress and lysosomal activity

**Figure 3:**
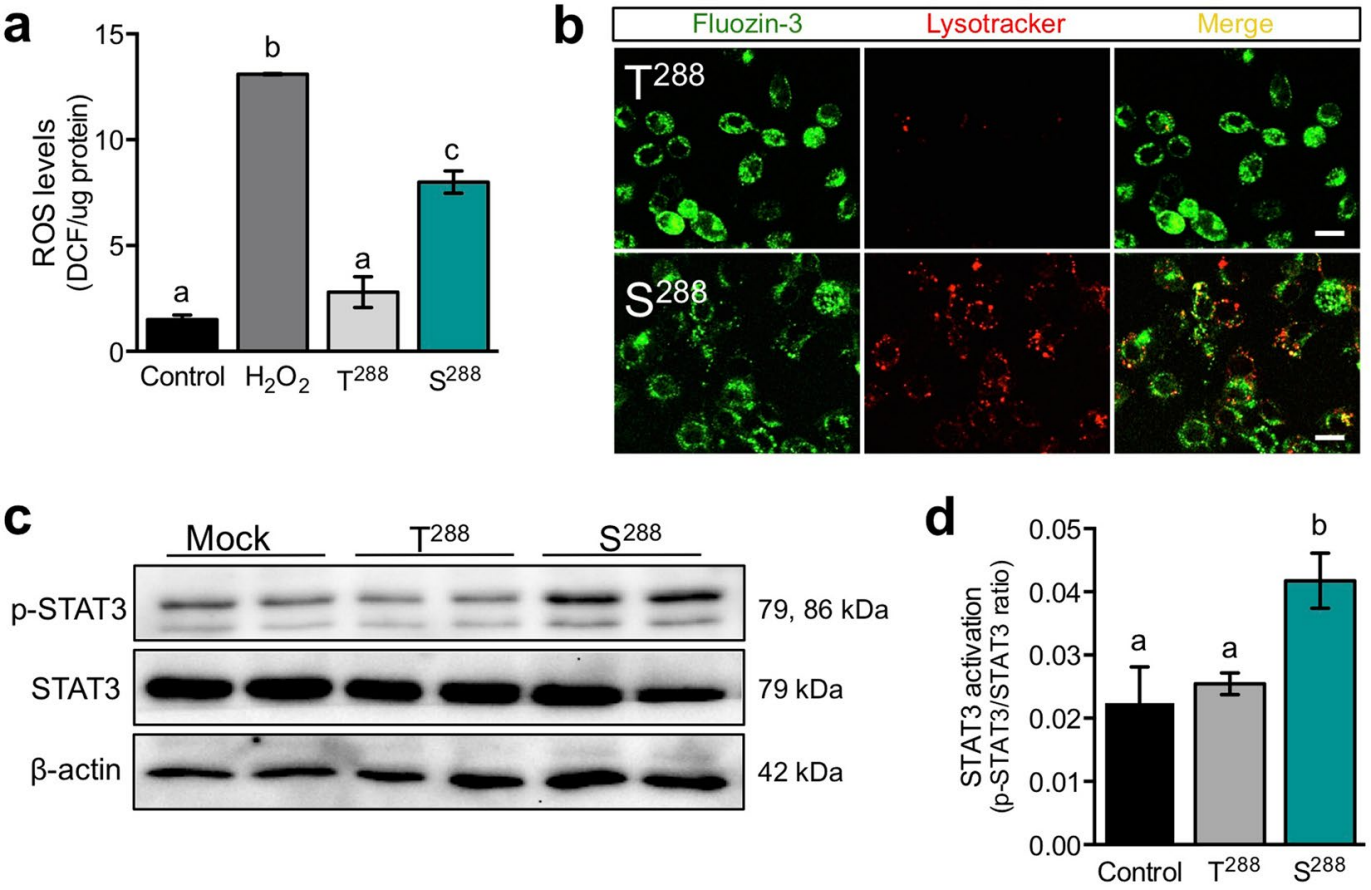
Cells expressing the S^288^ variant of ZnT2 have increased oxidative stress, lysosomal activity and STAT3 activation. (**a**) Assessment of reactive oxygen species (ROS) level in untransfected MECs (Control) or MECs transfected to express wild-type ZnT2 (T^288^) or the ZnT2 variant (S^288^). Cells treated with H_2_O_2_ (100 µM) were used as a positive control. Data represent mean DCF-HA fluorescence/µg of protein ± SD, from n = 6 samples/group; the experiment was repeated three times. Means with different letters are significantly different, p < 0.01. (**b**) Representative confocal images of FluoZin-3 (green) and Lysotracker Red (red) in MECs expressing T^288^ or S^288^. Merged images (yellow) illustrate co-localized FluoZin-3 and Lysotracker Red. Note greater Lysotracker Red fluorescence in MECs expressing T^288^ compared with MECs expressing S^288^ (scale bar, 20 µm). (**c**) Representative immunoblots of p-STAT3 and total STAT3 in cell lysates from MECs expressing T^288^, S^288^, or mock-transfected (Mock) cells. β-actin served as a loading control. Cropped blots are displayed and full-length blots can be found in Supplementary Fig. S4a–c. (**d**) Quantification of STAT3 activation. Data represent mean p-STAT3/total STAT3 ± SD from n = 6 samples/genotype, from three independent experiments. Means with different letters are significantly different, p < 0.05.

Several studies show that ER stress can trigger oxidative stress^55^. To determine if oxidative stress was enhanced in MECs expressing this mutant form of ZnT2, we ectopically expressed T^288^ or S^288^ in MECs *in vitro* and measured ROS levels using DCFH-DA. Using this technique, we noted that ROS levels were significantly higher (~3 fold) in MECs ectopically expressing S^288^ compared with MECs expressing T^288^ (Fig. 3a). Several reports indicate that oxidative stress can induce lysosomal activation^56,57^, which is a critical component of early involution in the mammary gland^20,58^. In addition to ER localization, we previously showed that the S^288^ variant is enriched in lysosomes^12,13,20^. Herein, confocal imaging in live MECs documented that FluoZin-3 fluorescence (a fluorescent reporter for labile Zn)^59^ was partially co-localized with Lysotracker Red in MECs expressing S^288^ but not in MECs expressing T^288^ (Fig. 3b), confirming that localization of S^288^ to lysosomes leads to lysosomal Zn accumulation. Moreover, we noted intense Lysotracker Red fluorescence in MECs expressing S^288^ that was not evident in cells expressing T^288^, suggesting increased lysosomal activity in cells expressing the S^288^ variant. Furthermore, we measured activation of the pro-involution transcription factor STAT3, also known to be induced by oxidative stress in the mammary gland^31,60^, and found that STAT3 activation was significantly increased in S^288^-expressing MECs (Fig. 3c and d). Because we previously showed that ZnT2-mediated Zn accumulation into lysosomes in MECs activates lysosomal-mediated cell death and STAT3 activation during involution^12^, it is enticing to speculate that expressing the mutant form of ZnT2 may trigger precocious mammary gland remodeling. However, expression of T^288^S does not alter cell cycle^1^, nor have we noticed appreciable cell death in our experiments. There may be several explanations for this discrepancy. First, MECs used in these experiments endogenously express wild-type ZnT2, which is consistent with documentation of heterozygosity in women who harbor S^288^ in our previous study^1^. This could buffer cytotoxic lysosomal Zn accumulation enough to prevent cell death. Second, Zn secretion^1^ in cells expressing S^288^ is greater than in cells expressing T^288^, suggesting that additional pathways for Zn export that may prevent cytotoxic lysosomal Zn accumulation exist.

### Expression of S^288^ impairs MEC barrier function

**Figure 4:**
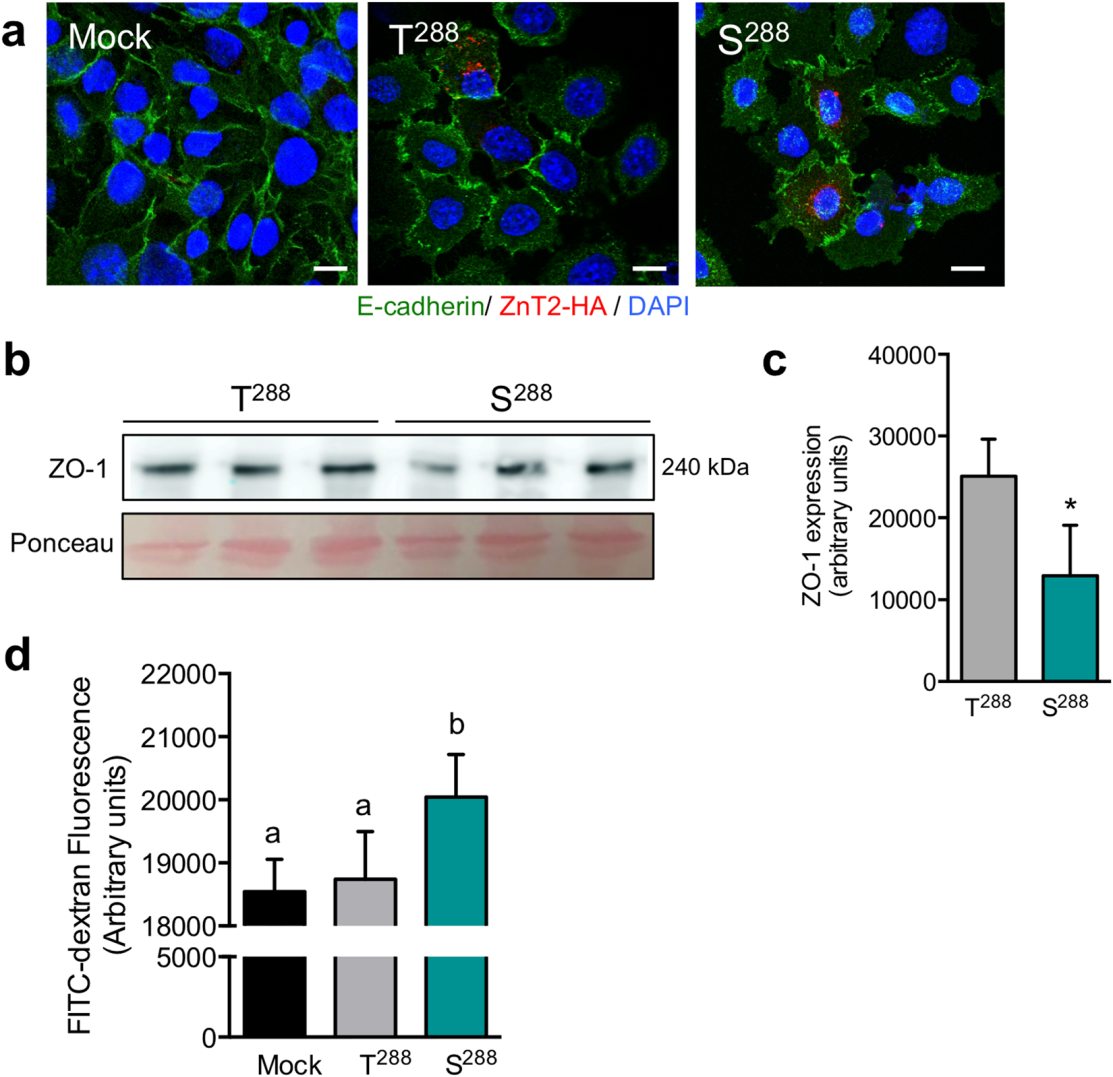
Barrier function is disrupted in MECs expressing the S^288^ variant of ZnT2. (**a**) Representative confocal images of E-cadherin (green) and ZnT2-HA (red) in MECs transfected to express wild-type ZnT2 (T^288^) or the ZnT2 variant (S^288^). Nuclei were counterstained with DAPI (blue; scale bar, 10 µm). (**b**) Representative immunoblot of zonula occludens-1 (ZO-1) in total cell lysates from MECs expressing T^288^ or S^288^. Ponceau staining served as a loading control. Cropped blots are displayed and full-length blots can be found in Supplementary Fig. S5a,b. (**c**) Quantification of ZO-1 expression. Data represent mean ZO-1 signal intensity ± SD from n = 3 samples/genotype; p < 0.05*. (**d**) Assessment of barrier function in cells expressing T^288^ and S^288^. Data represent mean FITC-dextran fluorescence (arbitrary units) ± SD, from n = 3 samples/genotype; the experiment was repeated two times. Mock represents mock-transfected MECs. Means with different letters are significantly different, p < 0.05.

Mammary epithelial cell polarity and barrier integrity are critical for optimal lactation^61^. Recently, we reported that the loss of ZnT2-mediated Zn transport disrupts E-cadherin and zonula occludens-1 (ZO-1) localization to establish intercellular junction formation^19^. E-cadherin is a transmembrane protein and a major contributor to epithelial integrity along with its associated cytosolic proteins α-catenin, β-catenin, and p120 catenin. E-cadherin loss leads to disruption in epithelial polarity and organization thus playing an essential role in MEC survival and function^62^. Zonula occludens-1 is a peripheral membrane protein that links integral tight junction proteins (i.e., occludens and claudins) to the actin cytoskeleton. Differentiation of MECs with prolactin reduces Erk1/2 signaling and leads to apical/basolateral polarity and tight junction barrier formation with E-cadherin localized basolaterally, and ZO-1 localized apically^63^. We previously reported that women harboring S^288^ have elevated milk sodium levels^1,24,64^, which has been linked to the disintegration of tight junctions that occurs as a result of oxidative stress and during involution^24,64^. To determine if tight junction barrier was compromised in MECs expressing S^288^, we assessed localization of E-cadherin, the expression of ZO-1, and measured transepithelial transport of FITC-dextran across a monolayer of MECs cultured in Transwells®. Studies using confocal imaging were inconclusive regarding potential defects in E-cadherin localization in MECs expressing S^288^. We noted that following prolactin treatment of sub-confluent MECs, E-cadherin staining was evident at the cell surface in MECs expressing both S^288^ and T^288^ (Fig. 4a), particularly where there was intercellular interaction. We next assessed effects of expressing S^288^ on ZO-1 expression and found that MECs expressing S^288^ had ~50% reduction in ZO-1 expression (Fig. 4b and c). A recent report in MDCK cells found that ZO-1 attenuation reduces polarization and tight junction assembly^65^, suggesting that MECs expressing S^288^ should have greater permeability. To address this question, MECs were transfected for 24 h to express S^288^ or T^288^, and then 5 × 10^5^ MECs were cultured in transwells for ~7 days until the transepithelial resistance (TEER) stabilized in untransfected control cells^66^. Following TEER stabilization, MECs were treated with prolactin for 48 h and the paracellular transport of small FITC-dextran molecules (~3 kDa) was measured. We found that MECs expressing S^288^ had a small but significant increase in transport of FITC-dextran (Fig. 4d). These data suggest that the loss of ZO-1 in MECs expressing S^288^ may lead to decreased interactions between other tight junction proteins (e.g., occludens and claudins) and the cytoskeleton, leading to a weakened barrier function, which is consistent with observations of higher milk sodium levels in women harboring T^288^S^1^. A small change in paracellular transport in S^288^-expressing MECs would be expected given that women harboring S^288^ were able to produce milk. However, it is important to note that milk volume and other lactation or infant outcomes were not assessed in our previous study.

### Substitution of serine at position 288 results in the hyperphosphorylation of ZnT2

**Figure 5:**
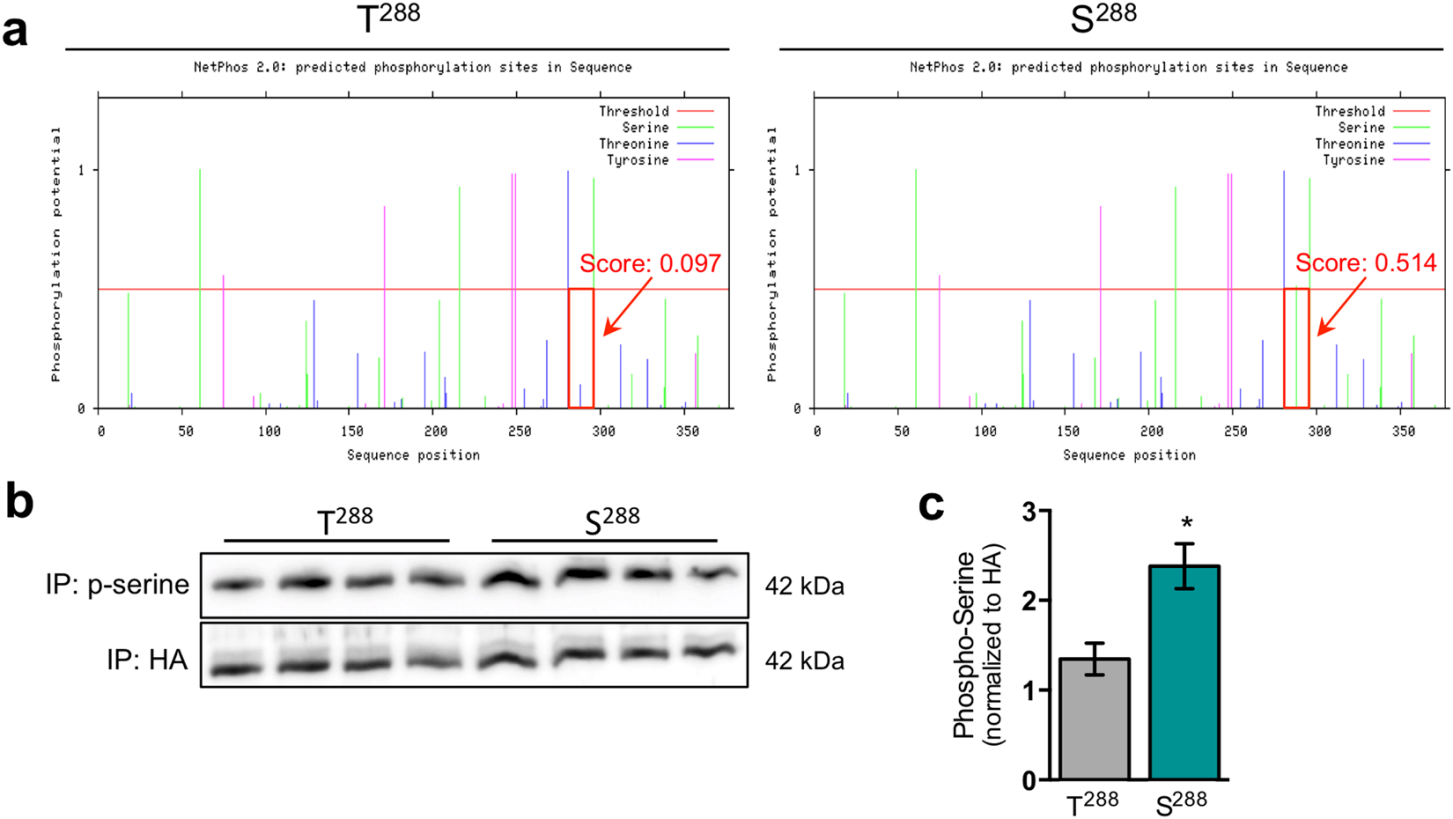
Substitution of serine for threonine at position 288 (S^288^) in ZnT2 leads to ZnT2 hyperphosphorylation. (**a**) Graphical representation of potential phosphorylation sites (serine, threonine and tyrosine) in wild-type ZnT2 (T^288^; left) and ZnT2 variant with a threonine to serine substitution (S^288^; right) as inferred from NetPhos 2.0. Green line represents potential phosphorylated serine residues; blue line represents potential phosphorylated threonine residues; pink line represents potential phosphorylated tyrosine residues; red horizontal line indicates threshold for modification potential; score indicates predicted phosphorylation potential score. (**b**) Representative immunoblot of phosphorylated serine in immunoprecipitates (IP) from MECs expressing T^288^ or S^288^. HA was used as normalization and input control. Cropped blots are displayed and full-length blots can be found in Supplementary Fig. S6a,b. (**c**) Quantification of serine phosphorylation. Data represent mean p-serine/HA ratio ± SD, n = 6 samples/genotype, from two independent experiments; p < 0.05*.

A key question that arises is why S^288^ is mis-localized to the ER and lysosomes. Several studies demonstrate that serine phosphorylation of transmembrane proteins is an ER retention signal^67,68^ and is associated with aggregate formation^69,70^ or oligomerization^71^, retaining them in the ER. Topology prediction of ZnT2 using ProteinProter (http://wlab.ethz.ch/protter/) displays six transmembrane domains with both N- and C-termini on the cytoplasmic side of the membrane. Because amino acid 288 resides in the cytoplasmic region, one would predict that it would be exposed to various kinases that are active in the cytoplasm of MECs, such as protein kinase C (PKC)^72^. Importantly, previous studies from our lab reveal that ZnT2 localization is regulated by post-translational (de)phosphorylation^12^, therefore we hypothesized that substitution of serine for threonine at amino acid 288 would affect ZnT2 phosphorylation and sub-cellular localization. We first compared the phosphorylation potential of T^288^and S^288^ using NetPhos 2.0 and found that a serine substitution at amino acid 288 had greater phosphorylation potential compared with the threonine in the wild-type ZnT2 sequence (Fig. 5a). To empirically confirm this, we assessed serine phosphorylation by immunoprecipitating ZnT2^S288^ and ZnT2^T288^ and immunoblotting for phospho-serine (Fig. 5b and c). Indeed, we found that the S^288^ variant had significantly greater serine phosphorylation compared to T^288^, indicating that the S^288^ variant is hyperphosphorylated. This suggests that the aberrant sub-cellular Zn transport seen in many ZnT2 variants *in vitro*^1,4^ may be due to altered post-translational modifications of ZnT2. Studies to confirm phosphorylation using mass spectroscopy were not successful, as neither tryptic or glutamyl endopeptidase (Glu-C) digests were able to generate peptides containing the amino acid at position 288 (data not shown). Therefore, while rigorous studies to confirm that S^288^ is a regulated phosphorylation site are required, our data provide compelling evidence that substitution of serine for threonine at amino acid 288 alters the overall phosphorylation state of the protein.

**Figure 6:**
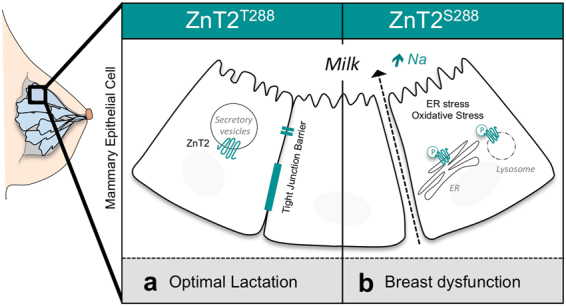
Model comparing MEC functions of wild-type ZnT2 (T^288^) and the ZnT2 variant (S^288^) during lactation. (**a**) Optimal lactation is achieved through tight regulation of milk secretion, MEC polarity and barrier integrity. During lactation, wild-type ZnT2 (T^288^) imports zinc into secretory vesicles in MECs, which is critical for secretory differentiation and secretory activation. (**b**) However, a common hyperphosphorylated ZnT2 variant (S^288^) is retained in the ER and lysosomes, leading to increased ER and lysosomal Zn accumulation, ER and oxidative stress, defects in tight junction formation and paracellular barrier formation, resulting in sodium leakage into milk.

In summary, our study provides structural and functional evidence that a common genetic variant in ZnT2 can affect key cellular functions in MECs. Our data indicate that mothers who harbor S^288^ express molecular factors in milk that reflect oxidative stress in the breast. The molecular defects revealed by our studies *in vitro* indicate that increased phosphorylation and mislocalization of S^288^ to the ER and lysosomes is associated with ER Zn accumulation, ER and oxidative stress, impaired paracellular barrier function, and lysosomal-mediated cell death (Fig. 6). Collectively, our study demonstrates that genetic variants in ZnT2 may have profound consequences on sub-cellular Zn pools and the molecular regulation of MEC function, which may lead to breast dysfunction and poor lactation performance in women. To our knowledge this is the first report that genetic variation may underlie sub-optimal lactation performance, and further studies to explore effects of genetic variation on breast function and infant health outcomes warrant consideration.

## Methods

### Breast milk analysis

The study was approved by the Institutional Review Board of the Pennsylvania State University and informed consent was obtained from all participants and/or their legal guardians. All experiments in this study were performed in accordance with relevant guidelines and regulations. Milk samples from women expressing two wild-type alleles (T^288^) and women harboring the ZnT2 variant (S^288^) were obtained from a previous study^1^. Milk samples (n = 4–5/group) were used to measure the percentage of milk fat by creamatocrit as previously described^73^. Frozen milk samples were thawed on ice and centrifuged at 2000 g for 15 minutes 4 °C to skim. Milk lactose was measured in skimmed milk samples using a lactose assay kit according to manufacturer’s instructions (Abcam, Cambridge, MA), and milk lactoferrin was measured in skimmed milk samples using ELISA kit according to manufacturer’s instructions (Abcam). Total milk protein concentration was measured by Dumas analysis^74^.

### Matrix metalloproteinase (MMP) activity

MMP-2 activity was assessed by gel zymography as previously described^18^. Briefly, milk samples were prepared by mixing with equal volume of Laemmeli sample buffer, and 10 µL of the prepared sample was loaded onto an SDS-PAGE gel containing 1% gelatin. Relative MMP activity was assessed by measuring gelatin lysis area (cleared band) and quantifying using Adobe Photoshop CS3.

### Generation of plasmid DNA construct

The full-length wild-type form of ZnT2 protein tagged with a C-terminal tandem hemagglutinin (HA; T^288^) was generated as previously described^75^. The ZnT2 variant substituting a threonine for a serine at amino acid 288 (S^288^) was generated as previously described^1^.

### Cell culture and *In Vitro* Expression of ZnT2 variant

Mouse MECs (HC11 cells) were a gift from Dr. Jeffery Rosen (Baylor College of Medicine, Houston, TX) and used with permission of Dr. Bernd Groner (Institute for Biomedical Research, Frankford, Germany). Cells were maintained in growth medium (RPMI 1640 supplemented with 10% fetal bovine serum, 5 μg/mL insulin, 10 ng/mL epidermal growth factor, and 50 mg/L gentamycin). Cells were plated in antibiotic-free growth medium in 6-well plates for protein expression and FRET imaging (on glass coverslips), or in 24-well plates for confocal imaging (on glass coverslips), or in 96-well plates for assays. Cells were transiently transfected with 4 μg (6-well plates), 0.8 μg (24-well plates) and 0.2 μg (96-well plates) of either T^288^ or S^288^ plasmid using Lipofectamine 2000 (Invitrogen) for 5 h according to manufacturer’s instruction. Transfected MECs were used for experiments 24 h later. Transfections were verified by immunoblotting with anti-HA antibody as described below.

### Immunoblotting

Skimmed milk (10 µL) or MEC lysates (20 µg of protein) were prepared in Laemmli sample buffer containing 100 mM dithiothreitol (DTT), electrophoresed and immunoblotted as previously described (3,10). The following antibodies were used: anti-4 hydroxynonenal (4-HNE, 1:1000; Abcam), anti-mucin-4 (1:200; Santa Cruz Biotechnology), anti-endoplasmin (1:1000; Abcam), anti-phospho-serine (1:1000; Sigma-Aldrich), anti-HA (1:1000; Roche Applied Scientific), anti-phospho-STAT3 (1:1000; Cell Signaling), anti-STAT3 (1:1000; Cell Signaling), anti-E-cadherin (1:100; Sigma) and anti-ZO-1 (1 µg/mL; Life Technologies). Antibodies were detected with horseradish peroxidase-conjugated anti- rabbit or anti-mouse IgG (GE Healthcare) or anti-goat IgG (Pierce). Membranes were stripped before re-probing with another antibody or β-actin (1:5000, Sigma-Aldrich) as loading or normalization controls where indicated. Protein was detected with SuperSignal Femto Chemiluminescent Detection System (Pierce) and imaged using digital imaging (FluorChem M, Cell Biosciences, USA). Band signal intensity was quantified using AlphaView software (ProteinSimple, San Jose, CA).

### Confocal imaging

Mouse MECs were immunostained as previously described^1^. Briefly, MECs were fixed with 4% paraformaldehyde for 10 min, permeabilized with 0.2% Triton X-100 for 10 min and then, stained with the following antibodies: anti-E-cadherin (1:50; Sigma) and anti-ZO-1 (5 µg/mL; Life Technologies). Primary antibodies were visualized using secondary antibodies conjugated with Alexa Fluor® 488 or Alexa Fluor® 568 (Life Technologies) and counterstained with DAPI nuclear stain (1 µg/mL; Molecular Probes). Cells were examined using a Leica Inverted Confocal Microscope SP8 (Leica Microsystems, Wetzlar, Germany).

### Transepithelial transport

Mouse MECs (5 × 10^5^ cells) were cultured on Transwell® cell culture inserts in growth medium for ~9 days until confluent. Transepithelial resistance (TEER) was used to monitor tight junction formation as previously described^66^, and experiments were conducted ~4 days post-TEER stabilization. Once confluent, MECs were cultured in differentiation medium (RPMI 1640 supplemented with 5 μg/mL insulin, 50 mg/L gentamycin, prolactin (1 µg/mL) and cortisol (2 mM) for 48 h. Fresh differentiation medium (0.5 mL) was added to the bottom chamber and differentiation medium containing fluorescein isothiocyanate-dextran (FD4; 0.2 mL containing 0.5 mg/mL) was added to the top chamber. Cells were incubated at 37 °C, and after 24 h the fluorescence (Ex 490 nm/Em 520 nm) in the medium in the bottom chamber was analyzed.

### Phosphorylation of ZnT2

Potential phosphorylation sites were predicted using NetPhos 2.0^76^, which uses an artificial neural network approach to predict the phosphorylation sites on serine, threonine and tyrosine residues. This program calculates a phosphorylation potential score of 0 to 1, where a value above the threshold of 0.5 indicates a potential phosphorylation site. The scores for ZnT2 incorporating either T^288^ or S^288^ were compared. Phosphorylation of ZnT2 was confirmed by immunoprecipitation followed by immunoblotting with phospho-serine antibody. Briefly, transfected MECs were washed in ice-cold PBS and lysed with radioimmunoprecipitation (RIPA) buffer for 5 min on ice. Cells were scraped into microcentrifuge tubes and briefly sonicated on ice. Samples were centrifuged for 10 min at 14,000 g at 4 °C. Protein concentration of lysates was determined using the Bradford assay (Bio-Rad, Hercules, CA). Lysates were pre-cleared with Protein A-Agarose beads (Sigma-Aldrich, St. Louis, MO) for 1 h and then incubated with anti-HA antibody for 3 h followed by incubating with Protein A-Agarose beads (Sigma-Aldrich) for 1 h at 4 °C with rotation. Beads were pelleted by centrifugation at 10,000 g for 2 min and washed four times each in RIPA buffer. Following the final wash, sample buffer was added to the resin and proteins were eluted and denatured by heating at 95 °C for 5 min. Samples were vortexed and centrifuged at 10, 000 g for 5 min to pellet the resin. Supernatants were loaded on a 10% polyacrylamide gel and immunoblotted for phospho-serine and anti-HA as normalization control.

### Endoplasmic Reticulum (ER) Zinc Sensor, ER-ZAPCY1

The pcDNA-ER-ZapCY1 vector was generated by Dr. Amy Palmer^52^ and purchased from Addgene (Cambridge, MA). ER-ZAPCY1is a high affinity Zn sensor targeted to the ER that is sandwiched between two fluorescent proteins, cyan fluorescent protein (CFP) and yellow fluorescent protein (YFP). Zinc binding induces a conformational change leading to an increase in fluorescence resonance energy transfer (FRET) from CFP to YFP^52^. To first confirm the localization of ER-ZAPCY1in our system, MECs were plated on glass coverslips in a 24-well plate and transfected with ER-ZAPCY plasmid (0.8 µg/well) using Lipofectamine 2000 as described above. After 24 h, MECs were fixed with 4% paraformaldehyde for 10 min, permeabilized with 0.2% Triton X-100 for 10 min and then ER-ZAPCY1(anti-GFP antibody; 1:50, Sigma-Aldrich) and calnexin (1:50; Abcam) were detected. Antibodies were visualized with Alexa Fluor® 488 or Alexa Fluor® 568 (Life Technologies) and counterstained with DAPI nuclear stain (1 µg/mL). Slides were examined using the Leica Inverted Confocal Microscope SP8 (Leica Microsystems). In subsequent experiments, MECs were co-transfected with T^288^ or S^288^ and ER-ZAPCY1 as described above, and ER-ZAPCY1 and ZnT2-HA (using anti-HA antibody; 1:100, Roche Applied Scientific) were detected then visualized with Alexa Fluor® 488 or Alexa Fluor® 568.

### FRET Analysis

HC11 cells were transfected to express T^288^ or S^288^ together with ER-ZAPCY as described above, and imaged using a Leica DMI 6000B inverted automated fluorescence microscope equipped with Hamamatsu ORCA-flash 4 Camera. Images were captured at 20 sec intervals to minimize photobleaching. At each time point, CFP, YFP and FRET images were collected using CFP (Ex 438 nm/Em 483 nm), YFP (Ex 500 nm/Em 542 nm), and FRET (Ex 438 nm/Em 542 nm) filter cubes at room temperature with the 40× oil objective (N.A.1.35; Leica) and processed using Slidebook 6.0 software (Intelligent Imaging Innovations). Calculation of three-channel corrected FRET values used the formula:

1 $${{\rm{FRET}}}_{{\rm{C}}}={{\rm{I}}}_{{\rm{DA}}}-\mathrm{Fd}/\mathrm{Dd}\ast {{\rm{I}}}_{{\rm{DD}}}-\mathrm{Fa}/\mathrm{Da}\ast {{\rm{I}}}_{{\rm{AA}}}$$

in which I_DD_, I_AA_ and I_DA_ are the intensities of background-subtracted CFP, YFP and FRET images, respectively, F_C_ is the corrected energy transfer, Fd/Dd is the measured bleed-through of CFP across the FRET filter (0.457), and Fa/Da is the measured bleed-through of YFP across the FRET filter (0.19). To minimize the variation caused by the different expression level of the ER-ZAPCY probe, FRET_C_ was normalized to the intensity of background-subtracted CFP:

2 $${\rm{R}}={{\rm{FRET}}}_{{\rm{C}}}/{{\rm{I}}}_{{\rm{DD}}}$$

To obtain the basal FRET signals (R), MECs were imaged in HEPES-buffered Hank’s Balanced Salt Solution (HHBSS) for 3–4 time points. To obtain the minimum FRET signals (R_min_), MECs were treated with TPEN (100 µM) and thapsigargin (2 µM). To obtain maximum FRET signals (R_max_), MECs were treated with Zn pyrithione (100 µM). For quantitative analysis, we utilized the FRET ratio to represent the data which was described by Yan Qin^52^ using the formula:

3 $${\rm{FRET}}\,{\rm{ratio}}=({\rm{R}}-{{\rm{R}}}_{{\rm{\min }}})/({{\rm{R}}}_{{\rm{\max }}}-{{\rm{R}}}_{{\rm{\min }}})$$

### Reactive Oxygen Species (ROS) Assay

Cell permeable 2, 7-dichlorodihydrofluoroscein diacetate (DCFH-DA) (Invitrogen) was used to measure ROS production. Cells were plated on 96-well plate and transfected to express either T^288^ or S^288^ as described above. Twenty-four h post-transfection, MECs were rinsed with PBS, pH 7.4, and treated with DCFH-DA (10 μM) for 1 h at 37 °C. The fluorescence of DCF (Ex 520 nm/Em 495 nm) was measured and protein concentration was determined by Bradford assay. Fluorescence measurements were normalized to total protein concentration (signal/μg of protein). As a positive control, MECs were pre-treated with H_2_O_2_ (100 μM) for 30 min.

### Statistical Analysis

Results are presented as mean ± standard deviation (SD). For human milk experiments, the expected mean and standard deviation of key oxidative stress proteins (mucin-4 and lactoferrin) assessd in our laboratory in healthy women with two WT SLC30A2 alleles is 1+/−0.25. Therefore, the minimum sample size needed to detect a 50% difference with a power of 0.85 and an alpha of 0.05 was 4 samples/group. For cell experiments, all samples were analyzed in at least duplicate and all experiments were repeated at least twice with independent samples (specific parameters are included in each Figure legend). Statistical comparisons were performed using two-tailed Student’s *t*-tests for 2 groups and one-way ANOVA with Bonferroni’s post-hoc test for multiple comparisons (Prism GraphPad, Berkeley, CA). Statistical significance was demonstrated at p < 0.05.

### Data availability

All data generated or analysed during this study are included in this published article (and its Supplementary Information files).

## Electronic supplementary material

Supplemental Figures
