# Supplementary material for: A genetic variant in SLC30A2 causes breast dysfunction during lactation by inducing ER stress, oxidative stress and epithelial barrier defects
Source: Sci Rep. 2018 Feb 23;8:3542. doi: 10.1038/s41598-018-21505-8 (PMC5824919; doi:10.1038/s41598-018-21505-8)

A genetic variant in *SLC30A2* causes breast dysfunction during lactation by inducing ER stress, oxidative stress and epithelial barrier defects

Sooyeon Lee[1], Yandong Zhou[1], Donald L. Gill[1], and Shannon L. Kelleher*[1, 2, 3]

Departments of [1]Cellular and Molecular Physiology, [2]Pharmacology and [3]Surgery, Penn State Hershey College of Medicine, Hershey, PA, 17033

[a] Present address: Department of Medicine, Stanford University, Stanford, CA 94305
[b] Present address: Department of Biomedical and Nutritional Sciences, University of Massachusetts Lowell, Lowell, MA, 01854
*Corresponding author shannon_kelleher@uml.edu

SUPPLEMENTARY INFORMATION

Supplementary Table S1. Macronutrient concentrations in breast milk from women harboring wild-type ZnT2 (T^{288}) or the ZnT2 variant (S^{288}) .

Macronutrient (protein, lactose and fat) concentrations were measured in breast milk from women harboring wild-type ZnT2 (T^{288}) or the ZnT2 variant with a threonine to serine substitution (S^{288}). Protein (g/L) was measured by Dumas analysis, lactose (g/dL) was measured using Lactose Assay kit, and fat (%) was determined by creamatocrit. Data represent mean concentration ± SD from n=4-5 samples/group; p-value obtained by Student's t-test.

Supplementary Figure S1. Confirmation of ER-ZAPCY1 sensor localization in cultured MECs.

Representative confocal images of the ER Zn sensor, ER-ZAPCY1 (green) and the ER marker, calnexin (red). Merged images (yellow) illustrate co-localization of ER-ZAPCY1 and calnexin in MECs. Nuclei were counterstained with DAPI (blue). Note co-localization of ER-ZAPCY1 and calnexin, validating this ER sensor in our cell model.

Supplementary Figure S2. **Gel and immunblots relating to Figure 1 are shown.**

(**a**) Full-length zymography gel of MMP-2 activity.

(**b**) Membrane was cut in half prior to immunoblot to probe for Mucin-4 (top) and 4-HNE (bottom).

(**c**) Membrane was cut in half prior to immunoblot to probe for Endoplasmin.

(**d**) Replicate gel stained with Coomassie Blue.

Supplementary Figure S3. **Immunoblots relating to Figure 2 are shown.**

(**a**) Full-length blot probed for Endoplasmin.

(**b**) Membrane was cut in half and re-probed for β-actin.

Supplementary Figure S4. **Immunoblots relating to Figure 3 are shown.**

(**a**) Full-length blot probed for p-STAT3.

(**b**) Membrane was cut in half to re-probe for total STAT3 (top) and β-actin (bottom).

Supplementary Figure S5. **Immunoblots relating to Figure 4 are shown.**

(**a**) Full-length blot probed for ZO-1.

(**b**) Membrane stained with Ponceau.

Supplementary Figure S6. **Immunoblots relating to Figure 5 are shown.**

(**a**) Full-length blot probed for p-serine.

(**b**) Full-length blot probed for HA.

Supplementary Table S1

Macronutrients**	T^{288}	S^{288}	p-value
Protein (g/L)	17.2 ± 3.2	21.2 ± 4.2	0.1232
Lactose (g/dL)	7.1 ± 1.0	7.2 ± 2.4	0.9215
Fat (%)	8.1 ± 3.3	7.7 ± 1.8	0.8486

**Units are different for each macronutrient.

Supplementary Figure S1

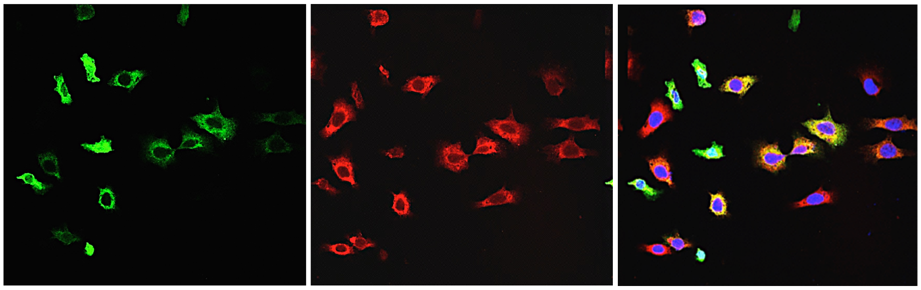

ER-ZapCY1 / Calnexin / DAPI / Merge

Supplementary Figure S2

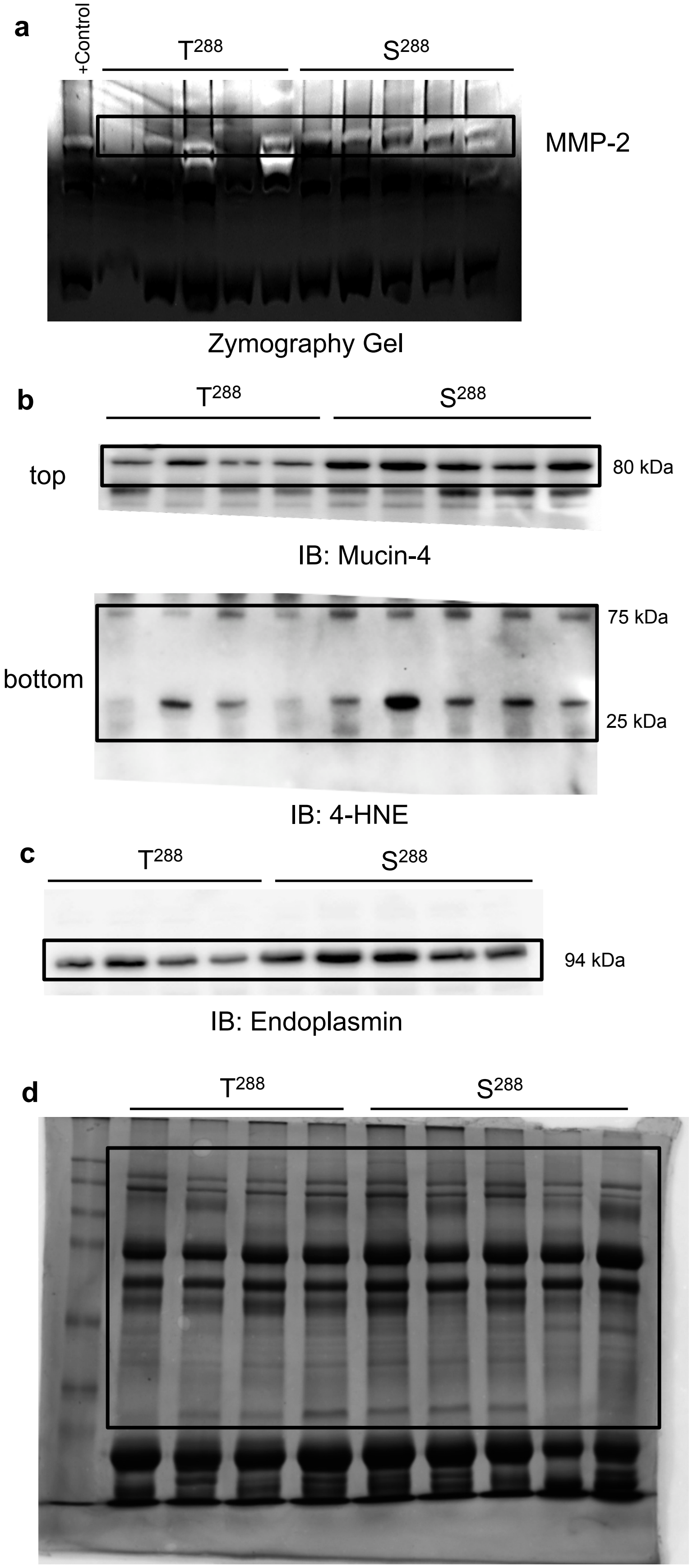

Supplementary Figure S3

a

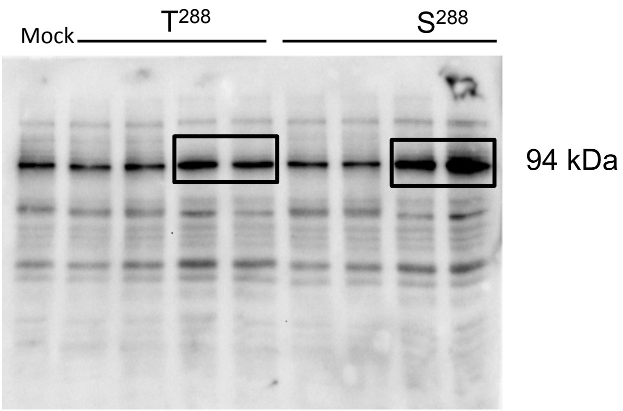

IB: Endoplasmin

b

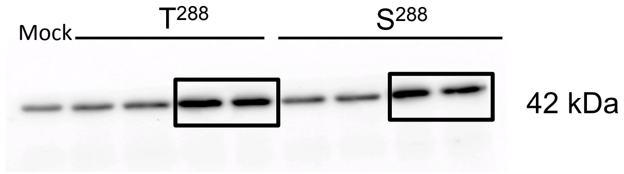

IB: β-actin

Supplementary Figure S4

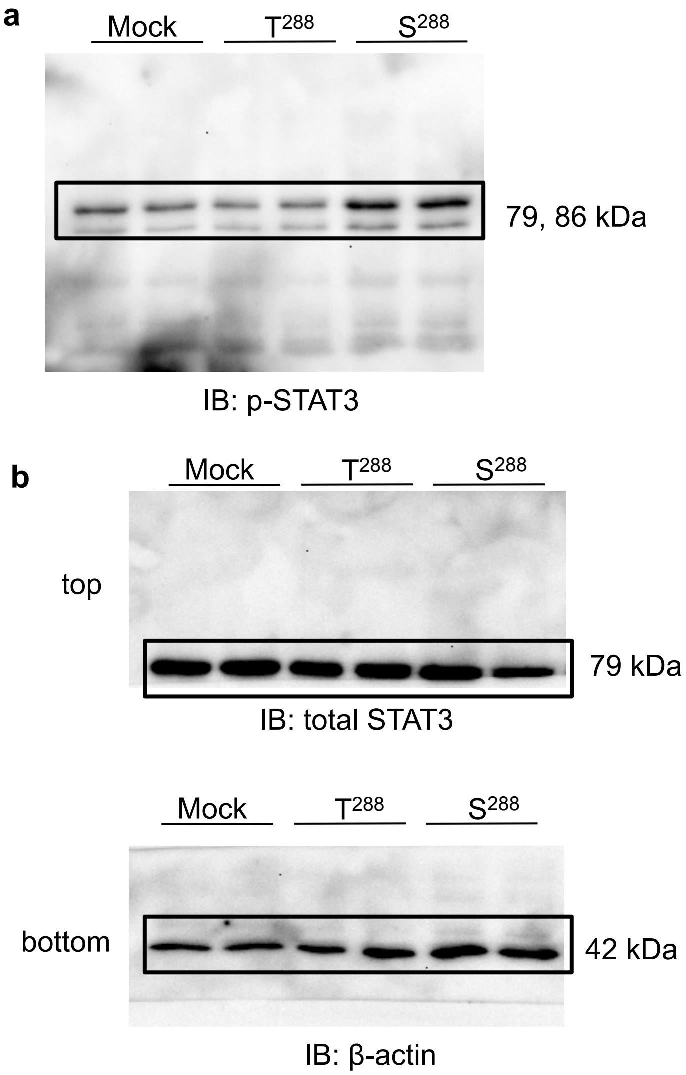

Supplementary Figure S5

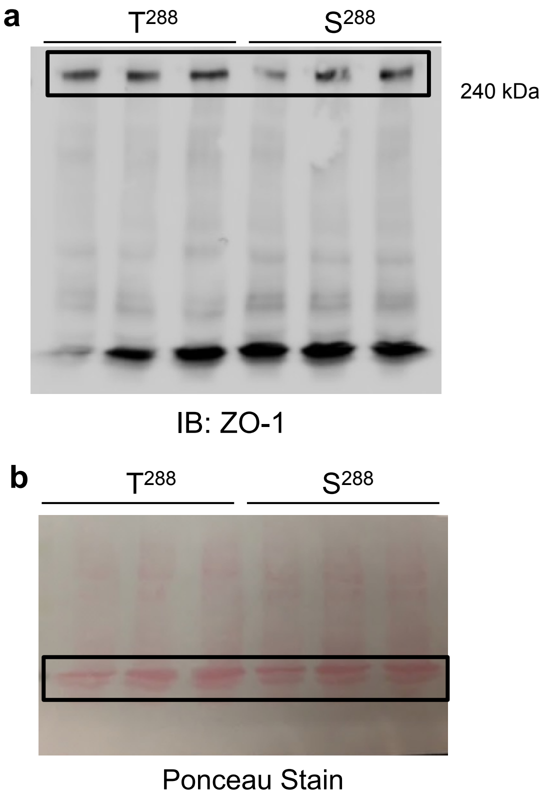

Supplementary Figure S6

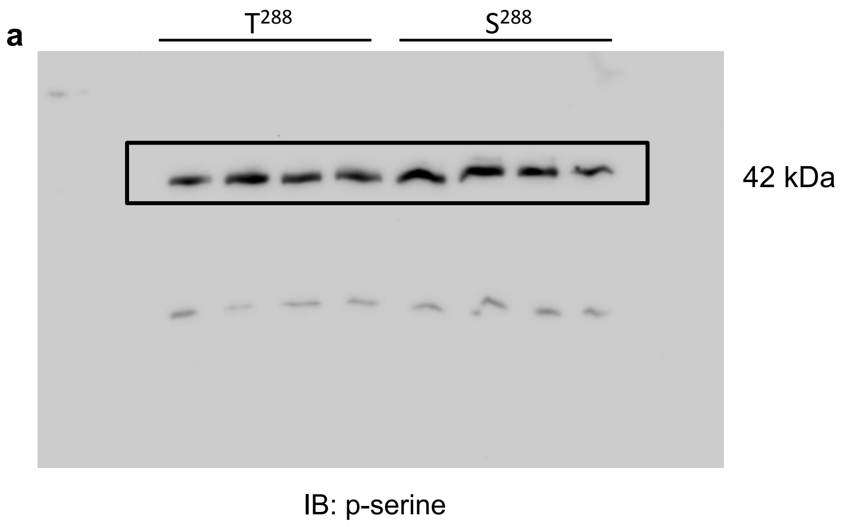

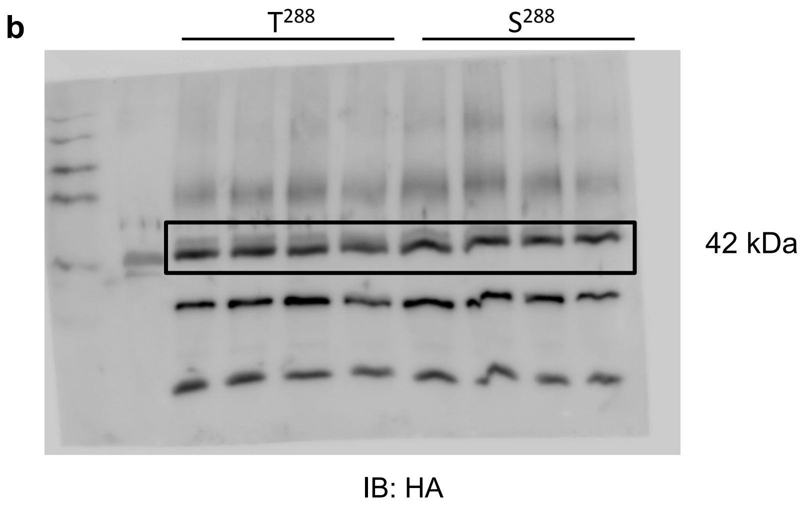

Supplement: Supplementary file 1 — Supplemental Figures [file 41598_2018_21505_MOESM1_ESM.pdf]
